# The Most Remarkable Clinic in the History of Dentistry

**Published:** 1900-03

**Authors:** Eugene S. Talbot


ARTICLE VIII.

### THE MOST REMARKABLE CLINIC IN THE HISTORY OF DENTISTRY.

REPORTED BY EUGENE S. TALBOT, M. D., D. D. S.

At the meeting of the American Medical Association, in Philade!phia in 1897, Dr. Bonwill read a paper before the Section on Stomatology upon the subject of "Cataphoresis; or, the Use of Electricity in Obtunding Sensitive Dentine." In closing the discusion Dr. Bonwill extended an invitation to a committee (appointed by the chairman) to visit his office at any time most convenient to them, to observe his method of producing anæsthesia by rapid breathing. The Chairman, Dr. R. R. Andrews, of Cambridge, Mass., appointed as a committee Drs. G. V. I. Brown, E. S. Talbot, M. H. Fletcher, A. W. Gillett and Geo. Eames. The committee met at the appointed hour, 9 o'clock, the following morning and, much to their surprise, found his spacious reception room filled with patients ranging in age from 14 to 78. These patients represented the extensive field of his practice. They were from Delaware, New York City and Philadelphia and had been summoned by telegraph and messengers. This illustrates the enthusiasm with which Bonwill entered into every undertaking. He had spent the entire night in this work. He had evidently enlarged, in his enthusiasm, the scope of investigation of the committee, since he proposed not only to illustrate his methods of practice at the present day in the younger patients, but also the results of his early practice in the elder.

Bonwill possessed perfect control of his patients. He would not operate for a patient who did not carry out his instructions to the letter. He frequently sent patients home and refused to operate, as they had not followed instructions given at a previous sitting. The cleanliness of all of his patients was remarkable. No inflamed gums or pus oozing from about the teeth was to be seen. The truth of his frequent statement "that he did not know what it was to have a case of 'pyorrhœa alveolaris' in his practice," was demonstrated by the mouths on exhibition. The committee had a very good opportunity, in the examination of the different patients, to see how he managed; the committee's questions to patients were answered in the presence of Bonwill without his knowledge, because of his deafness. The patients loved him, but said that they did not dare to visit his office for his services without complying with his instructions. Each patient was required to visit his office from four to six times a year and undergo a thorough inspection. If the brush was not being used in a proper manner or reaching certain localities they were instructed how to proceed. He gave his patients tooth brushes and tooth soap, so that there should not be the slightest excuse for unclean mouths. One of Bonwill's pet methods was the preparation of the teeth to secure getting wide spaces between them. The approximal cavities were partially prepared and red gutta-percha warmed and crowded in between the teeth; all approximal cavities were treated in this manner. The patient masticated upon this from six months to two or three years. By this method the teeth are wedged apart, making wide spaces, without inconvenience to the patient. The teeth were then contoured in such a manner that the occlusion was at the grinding edge, thus giving room for the alveolar process and firm foundation for the gum tissue.

Bonwill believed that the narrow alveolar process between the roots of the teeth failed to nourish the gum margin, thus causing interstitial gingivitis. Every operation upon the mouth was performed with the utmost skill, in a sense not understood by the average dentist. Bonwill was a master workman. His operations were perfection. The contour of the fillings, whether gold or amalgam, conformed to the movement of the jaws and the occluding teeth. Although Bonwill was an expert gold operator, many of his operations were done with amalgam, as he did not believe in crown and bridge work. In this he made another strong point as to the prevention of so-called pyorrhea alveolaris and unhealthy mouths. Crown and bridge work being a source of chronic irritation to the gum margin and the roots of two or more teeth supporting and doing the work of many more, and accumulating filth. Bonwill, in place of this, would build up the approximal surface and sometimes an entire crown (by introduction of screws), and polish the margin around and under the gum in such a manner that irritation or decomposed food and other irritants could not take place. Amalgam fillings were not confined to the posterior teeth. In many cases the anterior teeth were filled and contoured with amalgam. In the case of one old gentleman both approximal surfaces of all superior and interior incisors, as well as the bicuspids and molars, contoured with amalgam, had been doing good service for over thirty years. This case was of unusual interest, since osteomalacia (senile absorption of the alveolar process) had taken place, exposing the necks of all the teeth. The gums, however, were healthy, and no pus was present. The cavity margins at the approximal surfaces were smooth and free from decay; here was a fine opportunity to note the effect of wide approximal spaces.

In the place of bridge work, Bonwill had invented a removable plate (with which the profession is familiar.) This little plate was so nicely adjusted that it could be worn as a single tooth, or four or five could be attached when necessary. Fivs or six of these were exhibited, showing different methods of adjustment in favorable and unfavorable cases. Bonwill's method of adjusting artificial dentures and their practicable application was demonstrated in many mouths. He certainly knew how to arrange and adjust artificial teeth to the welfare of his patients. Patients were exhibited who had been wearing these dentures many years, some of which were very difficult to adjust. All were well pleased, as well they might be.

A few words about the reception room and office are necessary to complete. No one but Bonwill could arrange such an office. His æsthetic taste enabled him to furnish and fit up an office which resembled an art room more than a reception room to a dental office. The moment a patient entered the room he forgot the object of his visit. Art furniture, works of art about the room and upon the walls produced all the effects of suggestion (intentionally) upon his senses. Stand or sit anywhere, art books and bric-a-brac lay in profusion. These were so arranged that many could be handled at will. So interested would the patient become that time and dread were swallowed up and forgotten. One of the most singular features of Bonwill's life was that he was not a believer in evolution. He believed that the equilateral triangle was the basis upon which God constructed mankind, way back in the dark ages, and upon that basis he would remain until eternity. As a singular contradiction of this belief his patients could examine a series of about thirty photographs of Bonwill in a convenient corner of the reception room, demonstrating his evolution from childhood to the very year of his death. In these he took a natural but illogical pride, since they illustrate the helpless child developing year after year grasping new ideas as time passed by, adding nerve association to nerve association until he became a genius. He was emphatically a man who bettered the world by living in it.—*Dental Brief.*

